# Damage-associated molecular patterns in vitiligo: igniter fuse from oxidative stress to melanocyte loss

**DOI:** 10.1080/13510002.2022.2123864

**Published:** 2022-09-26

**Authors:** Jingying Wang, Yinghao Pan, Guangmin Wei, Hanxiao Mao, Rulan Liu, Yuanmin He

**Affiliations:** Department of Dermatology, The Affiliated Hospital of Southwest Medical University, Luzhou, Sichuan, People’s Republic of China

**Keywords:** Vitiligo, oxidative stress, damage-associated molecular patterns, pathogenesis, High mobility group box 1 (HMGB1), Heat shock protein 70 (Hsp70), S100B, Adenosine triphosphate (ATP), Interleukin, Antimicrobial peptides (AMPs)

## Abstract

**Objectives:**

The pathogenesis of vitiligo remains unclear. In this review, we comprehensively describe the role of damage associated molecular patterns (DAMPs) during vitiligo pathogenesis.

**Methods:**

Published papers on vitiligo, oxidative stress and DAMPs were collected and reviewed via database searching on PubMed, MEDLINE and Embase, etc.

**Results:**

Oxidative stress may be an important inducer of vitiligo. At high oxidative stress levels, damage-associated molecular patterns (DAMPs) are released from keratinocytes or melanocytes in the skin and induce downstream immune responses during vitiligo. Treatment regimens targeting DAMPs can effectively improve disease severity.

**Discussion:**

DAMPs play key roles in initiating host defenses against danger signals, deteriorating the condition of vitiligo. DAMP levels in serum and skin may be used as biomarkers to indicate vitiligo activity and prognosis. Targeted therapies, incorporating HMGB1, Hsp70, and IL-15 could significantly improve disease etiology. Thus, novel strategies could be identified for vitiligo treatment by targeting DAMPs.

## 1. Introduction

**Figure 1.**
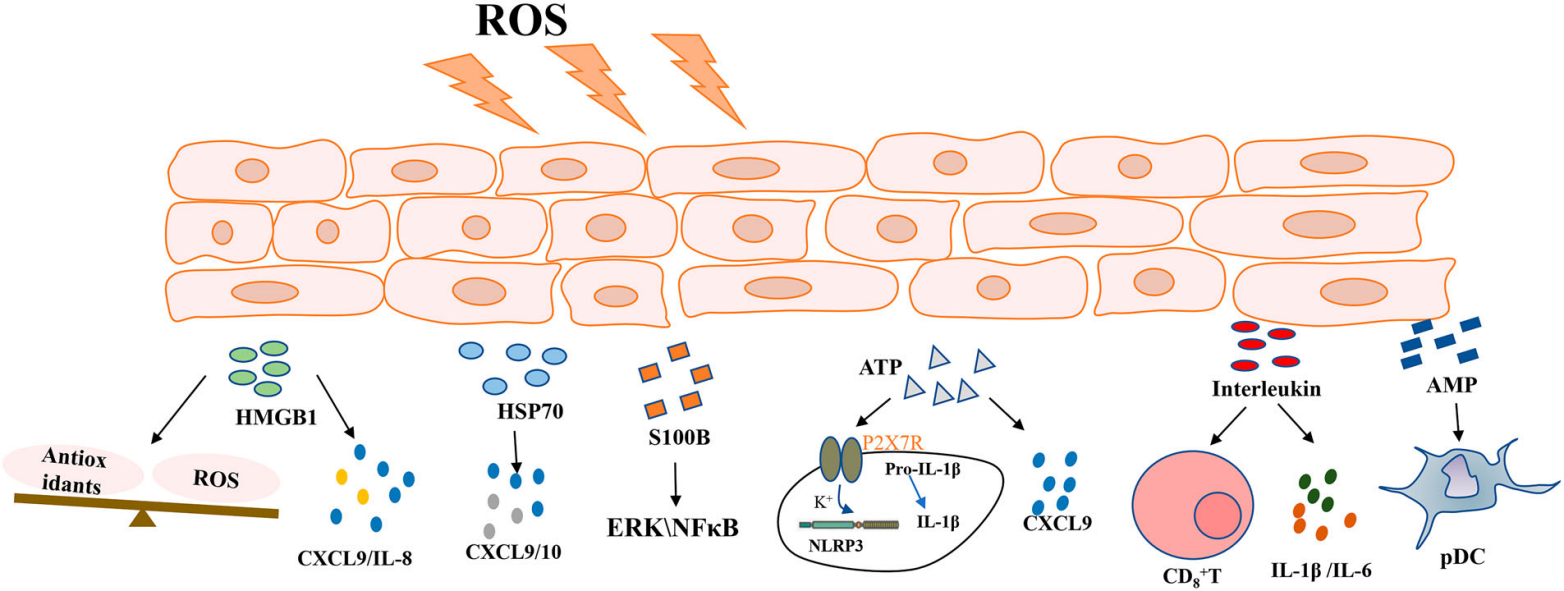
Oxidative stress promotes damage-associated molecular pattern secretion, including HMGB1, HSP70, S100B, ATP, the interleukins, and AMPs. These molecules increase cytokine release and accelerate melanocyte death. Abbreviations: HMGB1, high mobility group box 1; HSP70, heat shock protein 70; ATP, adenosine triphosphate; AMPs, antimicrobial peptides. ROS, reactive oxygen species; ERK, extracellular regulated protein kinase; NF-κB, nuclear factor kappa-B; NLRP3, the NOD-like receptor thermal protein domain associated protein 3; pDC, plasmacytoid dendritic cells.

Vitiligo is an autoimmune disease and is characterized by chronic depigmentation and milk-white lesion in the skin, with a 1% prevalence rate in the general population [1]. Although unaccompanied by distressing symptoms like pruritus or pain, vitiligo negatively affects the self-esteem and may cause anxiety or depression as it tends to occur in exposed skin areas [2]. Vitiligo is currently considered a genetic susceptibility disorder [3]; however, recent studies emphasized the important role of environmental factors in its etiology [4]. From disease initiation, oxidative stress plays significant roles in promoting vitiligo onset [5]. Also, epidermal melanocytes are particularly vulnerable to oxidative stress owing to their pro-oxidant status during melanin synthesis, resulting in melanocyte damage and self-antigen production [6]. In recent years, several studies reported that under adverse stimulus conditions, such as oxidative stress, damage-associated molecular patterns **(**DAMPs) were released from cells and participated in autoimmune disease onset by inducing sterile inflammation, eventually leading to vitiligo(Figure 1) [7].

## 2. Increased skin-based oxidative stress levels in patients with vitiligo

Mediated by disturbed redox homeostasis, oxidative stress is characterized by imbalanced pro-oxidant and antioxidant levels [8], and may be considered a crucial initiator of vitiligo [9]. Reactive oxygen species (ROS) generation is an instant intracellular response when cells are exposed to an external environment [10]. There are three major ROS: superoxide anion, hydrogen peroxide, and hydroxyl radical. Among the great variety of ROS, hydrogen peroxide (H_2_O_2_) has a pivotal role in the onset and progression of vitiligo. Excessive ROS production may also be simultaneously generated by melanogenesis and mitochondrial energy metabolism. Other than excessive ROS formation, aberrant ROS removal mechanisms may also account for increased ROS levels in the epidermis. Moreover, downregulated enzymatic or non-enzymatic defenses against oxidants in the epidermis, such as incapacitated catalase and glutathione peroxidase functions and decreased vitamin A and C levels also underpin ROS accumulation [11,12]. Another impaired antioxidant pathway in melanocytes and keratinocytes involves nuclear factor E2-related factor 2 (Nrf2)-antioxidant response element/heme oxygenase-1 (HO-1) activities [13,14].

ROS accumulation induces DNA damage and protein oxidation/fragmentation coupled with lipid peroxidation, thus reducing the function of these biological macromolecules [15]. In recent years, it was reported that oxidative stress in vitiligo lesions promoted DAMP release from surrounding cells and contributed to vitiligo occurrence [7].

## 3. DAMP characteristics

Danger sensing is a fundamental evolutionary feature enabling multicellular organisms to perceive potential threats, escape from dangerous situations, combat intruders, and repair physiological damage. Most molecules governing these processes are already present in cells (i.e. pre-formed DAMPs), therefore release is immediate, while others are neo-synthesized following injury. DAMPs, also known as alarmins, are endogenous and constitutively expressed proteins/peptides with immune-activating functions [7]. Most DAMPs are passively released from dead cells, but some are actively secreted to indicate an early state of sub-lethal cell stress [16]. In addition to physiological and homeostatic roles inside the cell, DAMPs also deliver, when exposed to the extracellular milieu, danger signals to the host, triggering local inflammatory responses [17]. Apart from their roles in disease initiation, DAMPs also amplify and sustain inflammatory processes, with notable roles in the pathogenesis of inflammatory conditions [18]. In addition to phagocyte activation and proinflammatory cytokine release, DAMPs are important links between the innate and adaptive immune systems, e.g. they activate immature dendritic cells (DCs) which process antigens. DAMPs present antigenic epitopes to naive T cells thereby inducing adaptive immune responses. It is largely accepted that DAMPs initiate immune responses by activating classical Pathogen Recognition Receptors (PRRs), which include not only toll-like receptors, but also multiple germline-encoded receptors, such as NOD-like receptors, retinoic acid-inducible gene I (RIG- I)-like receptors, C- type lectin receptors, and multiple intracellular DNA sensors [19].

In healthy individuals, DAMPs exert important intracellular roles by regulating DNA transcription, calcium homeostasis, cell proliferation, and differentiation. However, high extracellular DAMP levels are present in several pathologies and are related to disease severity in autoimmune conditions and inflammatory disorders, such as sepsis, psoriasis, traumatic brain injury, acute lung injury, inflammatory bowel disease, and arthritis [7,18,20]. In this review, we outline the relationships between different DAMPs and vitiligo pathogenesis.

## 4. Oxidative stress stimulates DAMP release and worsens vitiligo

As described, oxidative stress may be an important inducer of vitiligo. Also, melanocyte death in vitiligo skin is largely mediated by cytokines and chemokines [4]. Meanwhile, a number of DAMPs are found at high levels extracellularly in vitiligo, enhancing inflammatory responses that contribute to disease progression. These DAMPs include high mobility group box 1, heat shock protein 70, S100B proteins, adenosine triphosphate, the IL family, antimicrobial peptides and other biomolecules. Interestingly, DAMPs appear to bridge the gap between oxidative stress and some proinflammatory factors.

### 4.1. High mobility group box 1 (HMGB1)

HMGB1 is a highly conserved nuclear protein found in all cells. It is a multi-faceted protein exerting functions both inside and outside cells [21]. Intracellular HMGB1 regulates transcription repair and recombination by affecting chromosome structure [22]. Extracellular HMGB1 is actively released from immune cells (i.e. monocytes or macrophages) after stimulation with lipopolysaccharide, pro-inflammatory cytokines, or nitric oxide, and is passively released from dead or dying cells [22,23]. Extracellular HMGB1 acts as an alarmin which binds to multiple cell-surface receptors to stimulate the innate immune system and trigger inflammatory responses. Signaling pathways activated by HMGB-1 induce nuclear factor kappa-B (NF-κB) phosphorylation, which in turn generates several cytokines and chemokines, such as tumor necrosis factor-α (TNF-α), interleukin-1β (IL-1β), IL-6, macrophage inflammatory protein-1α, and transforming growth factor-β in many cells, including endothelial cells, fibroblasts, macrophages, monocytes, T cells, and B cells [24]. In fact, HMGB1 overexpression in pathological conditions, including septicemia, ischemia-reperfusion injury, arthritis, and cancers indicates key biological roles and clinical importance [25,26].

Vitiligo skin is in a state of high oxidative stress. Several studies reported that hydrogen peroxide (H_2_O_2_) stimulation promoted HMGB1 translocation and release from melanocytes. Furthermore, released HMGB1 inhibited the expression of Nrf2 and downstream antioxidant genes, aggravated oxidative stress, and induced melanocyte apoptosis. In fact, HMGB1 is overexpressed in blood samples and lesional specimens from patients with vitiligo [27]. Cui et al. reported that HMGB1 was released from the melanocyte nucleus in vitiligo perilesional skin. Highly expressed HMGB1 also promoted CXCL16 and IL-8 secretion from keratinocytes by binding to the receptor for advanced glycation end products and activating NF-κB and extracellular signal-regulated kinase signal pathways, thus mediating the chemotactic formation of CD_8_^+^ T cell migration and accelerating melanocyte death [27-29]. Additionally, HMGB1 promoted DC maturation in patients with vitiligo, contributing to oxidative stress-induced autoimmunity during the condition [27] [30]. In addition to melanocytes, oxidative stress also stimulates HMGB1 release from keratinocytes, drives melanocyte apoptosis and compensatory autophagy activation, and inhibits melanogenesis [29]. In short, HMGB1 worsens vitiligo by impacting melanocyte survival.

### 4.2. Heat shock protein 70 (Hsp70)

Hsp70 chaperones have several cell housekeeping activities, including newly synthesized protein-folding, polypeptide translocation into mitochondria, chloroplasts and the endoplasmic reticulum, protein complex disassembly, and protein activity regulation. Furthermore, Hsp70s prevent aggregation and promote the refolding of misfolded and denatured proteins, they solubilize aggregated proteins, and cooperate with cell degradation machinery to remove aberrant proteins and aggregates. Therefore, Hsp70s act as sentinel chaperones, guarding cells from the deleterious effects of a wide range of proteotoxic stresses [31]. Stressed cells halt mainstream protein synthesis in favor of Hsp and/or glucose-regulated protein synthesis pathways [32]. However, in addition to protective roles, Hsp70 promotes antigen uptake, and processes inflammatory responses via the major histocompatibility complex class I pathway and conventional class II pathway in antigen-presenting cells (e.g. DCs) leading to T cell subset activation [33]. Therefore, Hsp molecules exert cytoprotective effects. Once released into the extracellular environment, Hsp70 becomes an alarmin, induces immunity to secretory cells, and triggers pathological inflammatory diseases, such as type 1 diabetes, atherosclerosis, and rheumatoid arthritis, via immunomodulatory pathways [34-36]. In recent years, several studies have focused on Hsp mechanisms during vitiligo.

Diffuse and intense Hsp70 expression patterns occur in vitiligo skin when compared with healthy skin. The Hsp70 nuclear form is expressed in progressive forms of vitiligo [37,38]. Furthermore, in a study using 4-tert-butylphenol as a stress model, melanocytes were very sensitive to environmental stress stimulation and stimulated PIG3V (human vitiligo melanocyte cell line) to secrete Hsp70 [39]. In turn, Hsp70 induced membrane TNF-related apoptosis-inducing ligand expression and activated DC effector functions toward stressed melanocytes. *In vitro* studies revealed that the plasmacytoid dendritic cells (pDCs) expressing Hsp70 receptor Lox-1 (lectin-like oxidized low-density lipoprotein-receptor-1) aggregated Hsp70. Exogenous Hsp70 induced pDC activation and increased exogenous DNA uptake. Furthermore, Hsp70 potentiated DNA-induced interferon-α (IFN-α) production by pDCs, inducing CXCL9 and CXCL10 expression in keratinocytes and finally leading to melanocyte death [40].

In the vitiligo model of depigmentation which was formed by gene gun vaccination, the inclusion of human and mouse-derived inducible Hsp70 (HSP70i) in the vaccination protocol increased and accelerated depigmentation processes and was accompanied by the induction of lasting humoral responses to Hsp70 [41]. Jeffrey *et al.* reported that strong and lasting skin depigmentation was not induced in Hsp70i knockout mice, and that *in vivo* cytolytic assays showed no cytotoxic T-lymphocyte activity, and an absence of T-cell infiltration to the skin and hair follicle melanocyte maintenance. This study demonstrated that Hsp70i was necessary and sufficient to accelerate depigmentation in vitiligo-prone Pmel-1 mice, and was accompanied by lasting phenotypic changes in DC subpopulations [42]. Moreover, research also showed that mutant Hsp70 reversed autoimmune depigmentation in vitiligo [43]. And remarkable repigmentation following mutant Hsp70iQ435A-encoding DNA treatment. Also, repigmentation was accompanied by an initial influx of T cells accompanied by increased CD4/CD8 ratios. Importantly, treatment did not interfere with melanoma immunosurveillance. Therefore, Hsp70 proteins are potential vitiligo promoters [44,45].

### 4.3. S100B

S100B proteins are implicated in wide number of intracellular and extracellular functions, including apoptosis regulation, proliferation, differentiation, migration, invasion, energy metabolism, calcium ion homeostasis, protein phosphorylation, and inflammation [46]. Some S100B proteins are also secreted and exert extracellular paracrine and autocrine functions [46,47]. After immune cell damage or activation, S100B proteins are released into the extracellular space where they regulate immune and inflammatory processes. They act as DAMP molecules to activate both immune and endothelial cells by binding to toll-like receptors and receptors for advanced-glycation end products [48]. As some S100B proteins are easily identified in body fluids, they are used as biomarkers to detect specific diseases, where increased expression levels indicate pathological conditions [49].

S100B is a DAMP protein expressed in melanocytes and is proposed as a marker of melanocyte cytotoxicity. Serum S100B levels, in patients with active non-segmental vitiligo, were significantly increased and correlated with affected body surface areas, suggesting its potential as a vitiligo biomarker [50,51]. *In vitro* studies using repeat freeze–thaw procedures identified intracellular S100B upregulation in normal and vitiligo melanocytes prior to its extensive release into the circulation. This phenomenon could explain increased S100B serum levels in the active phase of vitiligo [52]. Once cells become damaged or necrotic, they secrete S100B into the circulation [53]. Extracellular S100B then activates extracellular signal-regulated protein kinase (ERK) and NF-κB by binding to their cell surface receptors [54]. In general, increased S100B levels are closely related to vitiligo activity, but their precise molecular mechanisms require further exploration.

### 4.4. Adenosine triphosphate (ATP)

ATP is a signal transmitter in non-adrenergic innervation stimulation, and its production is driven by the electron transport chain in the mitochondria [55]. ATP produced by glycolysis and oxidative phosphorylation usually exists at very high cellular concentrations, creating strong outward gradients, including chemical and elevator gradients, on negatively charged plasma membranes [56]. Extracellular ATP is a key DAMP molecule and is released into the extracellular medium during inflammation-induced injury to parenchymal cells, dying leukocytes, and activated platelets. Also, ATP directly activates the plasma membrane channel P2X7 receptor (P2X7R), leading to an intracellular influx of potassium ions, key triggers which activate the NOD-like receptor thermal protein domain associated protein 3(NLRP3) inflammasome. Simultaneously in the mitochondria, ROS and ATP production are coupled to regulate cellular redox reactions [57]. In healthy tissue stroma, levels of ATP and its metabolite adenosine are negligible, while their accumulation is significantly increased in inflammatory or tumor microenvironments [58].

Inflammasomes are key components of the host-defense system. They are essential inflammatory signaling platforms which detect injury mediators released during infection and tissue damage to activate inflammatory responses [59]. Genome-wide association analysis of patients with vitiligo showed the inflammasome pathway was involved in vitiligo pathogenesis, and that ATP was the most important link during inflammasome activation [59]. During vitiligo, the hyperoxidative stress state stimulates keratinocytes to release ATP outside the cell [60,61]. Treatment with ATP induces inflammasome and caspase-1 activation, and the production of active IL-1β and IL-18 forms via P2X7Rs in keratinocytes and melanocytes [62] [63-65]. High extracellular ATP levels also induce ROS production and cell death in melanocytes. Some vitiligo studies suggested that extracellular ATP, as a danger signal, activated the inflammasome pathway and increased the cutaneous chemotaxis of CD_8 _^+ ^T cells via CXCL9 [59]. Therefore, targeting ATP-P2X7 signaling could be a potential strategy for treating vitiligo. In other studies, vitiligo epidermal melanocytes displayed impaired ATP production when compared with healthy melanocytes, which further affected melanocyte migration abilities and led to pigment regeneration disorder [66,67]. In short, ATP deteriorates vitiligo by activating inflammasomes.

### 4.5. The IL family

IL-15 expression in the epidermis and serum of patients with vitiligo was significantly higher when compared with healthy controls, and highly correlated with H_2_O_2_ levels [68]. Oxidative stress promoted IL-15 and IL-15Rα expression, and also IL-15 trans-presentation by activating NF-κB signaling in keratinocytes, thereby contributing to effector memory T cell (CD_8 _^+ ^T_EM_) activation by IL-15-JAK-STAT signaling pathway [69]. Additionally, IL-15 was important for tissue resident memory T cell (T_RM_) production in viral infections and cutaneous lymphomas [70]. Moreover, IL-15 deficient-mice reportedly displayed impaired T_RM_ formation, while IL-15 promoted T_RM_ function *ex vivo*. Also, targeting IL-15 signaling using an anti-CD122 antibody reversed established vitiligo in mice. Short-term treatment with anti-CD122 also inhibited the T_RM_ production of IFN-γ and long-term treatment depleted T_RM_ from skin lesions [71].

Th17 cells are a subset of CD4^+^ T cells and secrete a variety of immunomodulatory molecules, including IL-17, which is increasingly implicated in the pathogenesis of several immune-mediated diseases [72]. Several studies reported that the frequency of peripheral blood Th17 cells and serum IL-17A levels in patients with vitiligo was higher than in healthy controls [73,74]. Vitiligo lesion biopsies also revealed Th17 cell infiltration [75]. An *in vitro* analysis also showed that the expression of microphthalmia-associated transcription factor (MITF) and downstream genes was downregulated in melanocytes post IL-17A treatment [76]. This treatment also induced morphological shrinking in melanocytes, resulting in decreased melanin production. In terms of the local cytokine network in the skin, IL-17A dramatically induced IL-1β, IL-6, and TNF-α production in skin-resident cells, such as keratinocytes and fibroblasts, to promote inflammation via a positive feedback loop [76].

IL-33 and ST2 expression were both increased in lesional skin, with serum IL-33 levels increased in patients with vitiligo. Further research showed that IL-33 was secreted by keratinocytes and functioned as an alarmin [77]. IL-33 also increased both IL-6 and TNF-α expression levels in primary keratinocytes and potentially induced melanocyte death by regulating cytokines in the cell microenvironment [78].

### 4.6. Antimicrobial peptides (AMPs)

AMPs are a diverse group of small bioactive proteins which are part of the body’s first line of defense against pathogen activation. They function by disrupting bacterial membranes, modulating immune responses, and regulating inflammation [79,80]. Some mammals have multiple cathelicidin genes, but in humans, cathelicidin antimicrobial peptide is the only cathelicidin gene which encodes the 18-kDa proprotein hCAP18. LL37 is one form of a mature cathelicidin peptide derived from hCAP18 by enzymatic cleavage with kallikreins in the epidermis [80]. LL37 modifies host immune responses, cell growth, migration, and differentiation [81]. Recently, abnormal LL37 expression was identified in several diseases such as psoriasis, atopic dermatitis, and rosacea [82,83]. Clinically, some patients will develop vitiligo in injured skin. Previous research also showed that keratinocytes co-expressed IFN, LL37, and MAVS in skin wounds and chronic inflammatory disease. LL37 enabled keratinocytes to produce IFN in response to double-stranded RNA from dying cells, and also LL37 functioned through MAVS-dependent activation of TBK1-AKT-IRF3 signaling pathway, meanwhile, IFN secreted by activated keratinocytes promoted DC maturation [84]. After interfering with LL37-DNA complexes, inflammation was significantly improved [85]. In vitiligo, IFN influenced chemokine secretion by surrounding keratinocytes and further recruited T cells through a positive feedback pathway, resulting in melanocyte death [86]. Atazadeh *et al.* reported that average blood LL37 levels in patients with vitiligo were significantly higher than in control groups, suggesting LL37 could be a potential threat during vitiligo; however, the mechanisms remain unclear [87].

## 5. Conclusion

DAMPs and their receptors play key roles in initiating host defenses against danger signals from innate and adaptive immune responses, deteriorating the condition of vitiligo (Table 1). DAMP levels in serum and skin may be used as biomarkers to indicate vitiligo activity and prognosis. Because most DAMPs are expressed locally and directly released during tissue injury, targeted therapies, incorporating HMGB1, Hsp70, and IL-15 could significantly improve disease etiology in animal models with abnormal inflammatory and autoimmune responses. Thus, novel strategies could be identified for vitiligo treatment by targeting DAMPs.

**Table 1.** 

Damage-Associated Molecular Patterns (DAMPS)	Biological activity	Serum levels in vitiligo	Role in vitiligo deterioration
**HMGB1**	Regulates transcription, repair, and recombination; stimulates innate responses; produces cytokines and chemokines [22]	Increased [29]	Inhibits the expression of Nrf2 and antioxidant genes; aggravates oxidative stress and induces melanocyte apoptosis; promotes CXCL16 and IL-8 secretion; mediates CD_8_ ^+^ T cell migration and accelerates melanocyte death; promotes dendritic cell maturation; autophagy activation; inhibits melanogenesis [27,29]
**HSP70**	Regulates protein activity; removes aberrant proteins; promotes antigen uptake, and induces inflammatory responses; activates T cell subsets [33]	Increased [37]	Activates dendritic cell effector functions [44]; induces CXCL9 and CXCL10 expression [40]
**S100B**	Regulates apoptosis, proliferation, differentiation, migration, invation, energy metabolism, protein phosphorylation, and inflammation [46]	Increased [51]	Biomarker of melanocyte cytotoxicity; activates extracellular signal-regulated protein kinase (ERK) and nuclear factor kappa-B(NF-κB) [52,53]
**ATP**	Signal transmitter, leads to an intracellular influx of potassium ions; a key trigger inducing NLRP3 inflammasome activation [65]	Not known	Activates inflammasomes and caspase-1; produces active IL-1β and IL-18 forms; induces reactive oxygen species production and melanocyte death; increases cutaneous chemotaxis of CD_8_ ^+^ T cells via CXCL9; affects melanocyte migration abilities and leads to pigment regeneration disorder [67]
**The interleukins**	Transmits information; activates and regulates immune cells; mediates T cell and B cell activation, proliferation, differentiation, and inflammatory response [70]	Increased [74]	Activates CD_8_ ^+^ T cells; induces IL-Iβ, IL-6, and tumor necrosis factor-α (TNF-α) production; induces melanocyte death [75,77]
**Antimicrobial peptides (LL37)**	Defense against pathogen activation; disrupts bacterial cell membranes, modulates immune response, and regulates inflammation [81]	Increased [87]	Requires further exploration
